# Testing average wind speed using sampling plan for Weibull distribution under indeterminacy

**DOI:** 10.1038/s41598-021-87136-8

**Published:** 2021-04-06

**Authors:** Muhammad Aslam

**Affiliations:** Department of Statistics, Faculty of Science, King Abdulaziz University, Jeddah, 21551 Saudi Arabia

**Keywords:** Climate sciences, Ecology, Mathematics and computing

## Abstract

The time truncated plan for the Weibull distribution under the indeterminacy is presented. The plan parameters of the proposed plan are determined by fixing the indeterminacy parameter. The plan parameters are given for various values of indeterminacy parameters. From the results, it can be concluded that the values of sample size reduce as indeterminacy values increase. The application of the proposed plan is given using wind speed data. From the wind speed example, it is concluded that the proposed plan is helpful to test the average wind speed at smaller values of sample size as compared to existing sampling plan.

## Introduction

Wind speed is an important parameter of wind energy. The meteorologists are interested to estimate the average wind speed for the next day, next month, or maybe for the next year, see^1^ for more details. In such a case, the meteorologists are interested to test the null hypothesis that the average wind speed is equal to the specified average speed versus the alternative hypothesis that average wind speed differs significantly. At the time of testing the hypothesis, it may not possible to record the average wind speed for a whole year for example. In this case, a random sample of days can be selected and the average wind speed can be recorded for those selected days only. The null hypothesis can be rejected if the daily average wind speed, say acceptance number of days, is more than or equal to the specified average wind speed during the given number of days. For example, let the specified average wind speed is 7mph and the average wind speed of 30 days is recorded. Let the meteorologists are decided to record the average wind speed for 10 days (acceptance number of days). Based on this information, the null hypothesis that the average wind speed is equal to 7 mph will be rejected if in 30 days, if the daily average wind speed in 10 days is less than 7 mph, otherwise, the alternative hypothesis is accepted. As the decision of daily average wind speed is taken on the basis of sample information, therefore, two types of errors are associated with testing the average wind speed. The probability that rejecting the null hypothesis when it is true is called the type-I error and the probability of accepting it when wrong is known as type-II error. The acceptance sampling plan can help meteorologists to choose the sample size of days and acceptance number of days that minimize both errors. The details about the acceptance sampling plans can be seen in^2,3^.

The wind speed data is recorded at random and follows the statistical distribution. Among other statistical distributions, the Weibull distribution has been applied widely for estimating and forecasting the wind speed. Akpinar et al.^4^ presented the statistical study for wind speed data. Yilmaz and Çelik^5^ discussed the statistical method to estimate wind speed. Ali et al.^6^ applied the Weibull distribution and Rayleigh distribution for the wind speed data. Arias-Rosales and Osorio-Gómez^7^ used the statistical analysis for estimating energy cost. The comparisons of wind speed distributions can be seen in^8–11^ used the Weibull distribution for modeling wind speed data. Campisi-Pinto et al.^12^ presented the statistical tests for surface wind speed. ul Haq et al.^13^ applied the Marshall–Olkin Power Lomax distribution for wind speed estimation. More applications of statistical techniques in analyzing the wind speed data can be read in^14–17^.

For estimating and forecasting, the statistical distributions under classical statistics can only be applied when the observations or the parameters are determined. Usually, the daily wind speed data is recorded in intervals. In this case, the statistical distributions under classical statistics cannot be applied. Alternately, the statistical methods using fuzzy logic can be applied for estimating purposes. Jamkhaneh et al.^18^ worked on the single sampling plan using a fuzzy approach. Jamkhaneh et al.^19^ discussed the effect of sampling error on inspection using a fuzzy approach. Sadeghpour Gildeh et al.^20^ proposed a single plan using fuzzy logic. Afshari and Sadeghpour Gildeh^21^ proposed the improved sampling plan using fuzzy logic. For details, the reader may refer to^22,23^.

The neutrosophic logic gives information about the measure of determinacy, and indeterminacy and measure of falseness, see^24^. Therefore, the neutrosophic logic is more efficient than the fuzzy logic and interval-based analysis. Later on, several authors worked on neutrosophic logic for various real problems and showed its efficiency over fuzzy logic, see, for example^25–30^. The idea of neutrosophic statistics was given using the idea of neutrosophic logic^31–33^. The neutrosophic statistics gives information about the measure of determinacy and measure of indeterminacy, see^34^. The neutrosophic statistics reduces to classical statistics if no information is recorded about the measure of indeterminacy. References^35–37^ proposed the acceptance sampling plans using the neutrosophic statistics. Aslam et al.^38^ proposed the time-truncated group plans for the Weibull distribution. Alhasan and Smarandache^39^ worked on neutrosophic Weibull and neutrosophic family of Weibull distribution.

The existing sampling plans based on classical statistics and fuzzy logic do not give information about the measure of indeterminacy. By exploring the literature and best of our knowledge, there is no work on a time-truncated plan for the neutrosophic Weibull distribution. In this paper, the neutrosophic Weibull distribution is introduced and applied for testing the daily average wind speed. The plan parameters for testing the hypothesis will be determined by minimizing type-I and type-II errors. It is expected that a smaller sample size is needed for testing the average wind speed using the proposed sampling plan.

## Methodology

In this section, the Weibull distribution under neutrosophic statistics is introduced. We will also present the design of the sampling scheme plan for testing the average wind speed under an indeterminate environment.

### Preliminaries

Suppose that $f\left({x}_{N}\right)=f\left({x}_{L}\right)+f\left({x}_{U}\right){I}_{N};{I}_{N}\epsilon \left[{I}_{L},{I}_{U}\right]$ be a neutrosophic probability density function (npdf) having determinate part $f\left({x}_{L}\right)$, indeterminate part $f\left({x}_{U}\right){I}_{N}$ and indeterminacy interval ${I}_{N}\epsilon \left[{I}_{L},{I}_{U}\right]$. Note that ${x}_{N}\epsilon \left[{x}_{L},{x}_{U}\right]$ be a neutrosophic random variable follows the npdf. The npdf is the generalization of pdf under classical statistics. The proposed neutrosophic form of $f\left({x}_{N}\right)\epsilon \left[f\left({x}_{L}\right),f\left({x}_{U}\right)\right]$ reduces to pdf under classical statistics when $I_{L} = 0$. Based on this information, the npdf of the Weibull distribution is defined as follows

1 $$f\left( {x_{N} } \right) = \left\{ {\left( {\frac{\beta }{\alpha }} \right)\left( {\frac{{x_{N} }}{\alpha }} \right)^{\beta - 1} e^{{ - \left( {\frac{{x_{N} }}{\alpha }} \right)^{\beta } }} } \right\} + \left\{ {\left( {\frac{\beta }{\alpha }} \right)\left( {\frac{{x_{N} }}{\alpha }} \right)^{\beta - 1} e^{{ - \left( {\frac{{x_{N} }}{\alpha }} \right)^{\beta } }} } \right\}I_{N} p;I_{N} \in \left[ {I_{L} ,I_{U} } \right]$$

where $\alpha$ and $\beta$ are scale and shape parameters, respectively. Note here that the proposed npdf of the Weibull distribution is the generalization of pdf of the Weibull distribution under classical statistics. The neutrosophic form of the npdf of the Weibull distribution reduces to the Weibull distribution when $I_{L} = 0$. The neutrosophic cumulative distribution function (ncdf) of the Weibull distribution is given by

2 $$F\left( {x_{N} } \right) = 1 - \left\{ {e^{{ - \left( {\frac{{x_{N} }}{\alpha }} \right)^{\beta } }} \left( {1 + I_{N} } \right)} \right\} + I_{N} ; I_{N} \in \left[ {I_{L} ,I_{U} } \right]$$

The neutrosophic mean of the Weibull distribution is given by

3 $$\mu_{N} = \alpha {\Gamma }\left( {1 + 1/\beta } \right)\left( {1 + I_{N} } \right);I_{N} \in \left[ {I_{L} ,I_{U} } \right]$$

The median life for the neutrosophic Weibull distribution is given by

4 $$\tilde{\mu }_{N} = \alpha \left( {{\text{ln}}\left( 2 \right)} \right)^{1/\beta } \left( {1 + I_{N} } \right);I_{N} \in \left[ {I_{L} ,I_{U} } \right]$$

### Methodology

The null and alternative hypotheses for the average wind speed are stated as follows:

$$H_{0} :\mu = \mu_{0} \;{\text{Vs}}{.}\;H_{1} :\mu \ne \mu_{0}$$

where $\mu$ is true average wind speed and ${\mu }_{0}$ is the specified average wind speed. Based on this information, the proposed sampling plan is stated as follows

*Step 1* Select a random sample of $n$ number of days and record the daily average speed for these selected days. Specify the number of days, say $c$, average wind speed ${\mu }_{0}$ and indeterminacy parameter ${I}_{N}\epsilon \left[{I}_{L},{I}_{U}\right]$.

*Step 2* Accept ${H}_{0}:\mu ={\mu }_{0}$ if daily average wind speed in $c$ days is more than or equal to ${\mu }_{0}$, otherwise, reject ${H}_{0}:\mu ={\mu }_{0}$.

The proposed sampling scheme is characterized by the three parameters $n$, $c$ and ${I}_{N}$, where ${I}_{N}\epsilon \left[{I}_{L},{I}_{U}\right]$ is considered as the specified parameter and set according to the uncertainty level. Suppose that ${t}_{0}=a{\mu }_{0}$ be the time in days, where $a$ is the termination ratio. The probability of accepting ${H}_{0}:\mu ={\mu }_{0}$ is given by

5 $$L\left( p \right) = \mathop \sum \limits_{i = 0}^{c} \left( {\begin{array}{*{20}c} n \\ i \\ \end{array} } \right)p^{i} \left( {1 - p} \right)^{n - i}$$

where $p$ is the probability of rejecting ${H}_{0}:\mu ={\mu }_{0}$ and obtained using Eqs. (2) and (3) and defined by

6 $$p=1-\left\{\mathrm{exp}(-{a}^{\beta }{\left(\mu /{\mu }_{0}\right)}^{-\beta }{{\left(\Gamma \left(1/\beta \right)/\beta \right)}^{\beta }\left(1+{I}_{N}\right)}^{\beta })\left(1+{I}_{N}\right)\right\}+{I}_{N}$$

where $\mu /{\mu }_{0}$ is the ratio of true average daily wind speed to specified average daily wind speed. Suppose that $\stackrel{\sim }{\alpha }$ and $\stackrel{\sim }{\beta }$ be type-I and type-II errors. The meteorologists are interested to apply the proposed plan for testing ${H}_{0}:\mu ={\mu }_{0}$ such that the probability of accepting ${H}_{0}:\mu ={\mu }_{0}$ when it is true should be larger than $1-\stackrel{\sim }{\alpha }$ at $\mu /{\mu }_{0}$ and the probability of accepting ${H}_{0}:\mu ={\mu }_{0}$ when it is wrong should be smaller than $\stackrel{\sim }{\beta }$ at $\mu /{\mu }_{0}=1$. The plan parameters for testing ${H}_{0}:\mu ={\mu }_{0}$ will be obtained such that the following two inequalities are satisfied.

7 $$L\left({p}_{1}|\mu /{\mu }_{0}=1\right)\le \stackrel{\sim }{\beta }$$

8 $$L\left({p}_{2}|\mu /{\mu }_{0}\right)\ge 1-\stackrel{\sim }{\alpha }$$

where ${p}_{1}$ and ${p}_{2}$ are defined by

9 $${p}_{1}=1-\left\{\mathrm{exp}(-{a}^{\beta }{{\left(\Gamma \left(1/\beta \right)/\beta \right)}^{\beta }\left(1+{I}_{N}\right)}^{\beta })\left(1+{I}_{N}\right)\right\}+{I}_{N}$$

and

10 $${p}_{2}=1-\left\{\mathrm{exp}(-{a}^{\beta }{\left(\mu /{\mu }_{0}\right)}^{-\beta }{{\left(\Gamma \left(1/\beta \right)/\beta \right)}^{\beta }\left(1+{I}_{N}\right)}^{\beta })\left(1+{I}_{N}\right)\right\}+{I}_{N}$$

The values of the plan parameters $n$ and $c$ for various values of $\stackrel{\sim }{\beta }$, $\stackrel{\sim }{\alpha }=0.10$, $a$ and ${I}_{N}$ are placed in Tables 1, 2, 3, 4, 5 and 6. Tables 1 and 2 are shown for the exponential distribution case. For exponential distribution, it can be seen that the values of $n$ decreases as the values of $a$ increases from 0.5 to 1.0. On the other hand for other the same parameters, the values of $n$ decreases as the values of $\beta$ increases. Note here that the indeterminacy parameter ${I}_{N}$ also plays a significant role in minimizing the sample size.

**Table 1:** The plan parameter when $\stackrel{\sim }{\alpha }=0.10;\beta =1$ and $a=0.50$.

$\stackrel{\sim }{\beta }$	$\mu /{\mu }_{0}$	${I}_{U}$ = 0				${I}_{U}$ = 0.02				${I}_{U}$ = 0.04				${I}_{U}$ = 0.05			
		$n$	$c$	$L\left({p}_{1}\right)$	$L\left({p}_{2}\right)$	$n$	$c$	$L\left({p}_{1}\right)$	$L\left({p}_{2}\right)$	$n$	$c$	$L\left({p}_{1}\right)$	$L\left({p}_{2}\right)$	$n$	$c$	$L\left({p}_{1}\right)$	$L\left({p}_{2}\right)$
0.25	1.1	1143	438	0.9012	0.2485	1091	433	0.9009	0.2478	1037	426	0.9008	0.2488	1010	422	0.9006	0.2493
	1.2	327	122	0.9011	0.2433	318	123	0.9049	0.2444	300	120	0.9014	0.2417	290	118	0.9006	0.2435
	1.3	165	60	0.9031	0.2414	159	60	0.9066	0.2451	154	60	0.9034	0.235	151	60	0.9082	0.2426
	1.4	104	37	0.9084	0.2474	98	36	0.9026	0.2412	97	37	0.9096	0.2429	93	36	0.9047	0.24
	1.5	72	25	0.9064	0.249	72	26	0.914	0.2493	67	25	0.9096	0.2491	66	25	0.9081	0.2438
	1.8	37	12	0.9085	0.2465	36	12	0.9037	0.2326	35	12	0.9108	0.2471	34	12	0.9079	0.2369
	2	33	10	0.9064	0.1889	32	10	0.9039	0.181	30	10	0.9206	0.2146	29	10	0.929	0.2353
0.1	1.1	1955	741	0.9008	0.0994	1867	733	0.9008	0.0991	1779	723	0.9012	0.0998	1737	718	0.9011	0.0998
	1.2	552	202	0.9015	0.0998	525	199	0.9005	0.0996	507	199	0.9024	0.0989	491	196	0.901	0.0994
	1.3	277	98	0.9017	0.0979	267	98	0.9052	0.0993	253	96	0.9011	0.0968	246	95	0.9016	0.0984
	1.4	175	60	0.9037	0.0972	169	60	0.9034	0.0945	166	61	0.9052	0.0901	155	58	0.9023	0.0973
	1.5	126	42	0.9084	0.0975	119	41	0.9036	0.0952	118	42	0.9035	0.0872	110	40	0.9048	0.0986
	1.8	65	20	0.9119	0.0975	60	19	0.9023	0.0955	58	19	0.9018	0.0925	57	19	0.9021	0.0918
	2	51	15	0.9189	0.0937	49	15	0.9218	0.0956	47	15	0.9262	0.0998	47	15	0.9156	0.0836
0.05	1.1	2544	960	0.9008	0.0499	2428	949	0.9002	0.0495	2311	935	0.9002	0.0498	2260	930	0.9003	0.0496
	1.2	722	262	0.9016	0.0495	689	259	0.9009	0.0492	663	258	0.9015	0.0482	639	253	0.9009	0.0499
	1.3	363	127	0.9034	0.049	345	125	0.9014	0.0485	333	125	0.9038	0.0482	322	123	0.9035	0.0496
	1.4	225	76	0.9019	0.0493	220	77	0.9037	0.0469	210	76	0.9006	0.0452	203	75	0.906	0.0498
	1.5	162	53	0.9054	0.0486	156	53	0.9088	0.0494	148	52	0.9058	0.0485	143	51	0.9011	0.0475
	1.8	84	25	0.9019	0.0441	81	25	0.9027	0.0432	80	26	0.9214	0.0493	76	25	0.9138	0.0487
	2	64	18	0.9018	0.0415	58	17	0.9016	0.0486	56	17	0.9018	0.0472	55	17	0.9025	0.047

**Table 2:** The plan parameter when $\stackrel{\sim }{\alpha }=0.10;\beta =1$ and $a=1.0$.

$\stackrel{\sim }{\beta }$	$\mu /{\mu }_{0}$	${I}_{U}$ = 0				${I}_{U}$ = 0.02				${I}_{U}$ = 0.04				${I}_{U}$ = 0.05			
		$n$	$c$	$L\left({p}_{1}\right)$	$L\left({p}_{2}\right)$	$n$	$c$	$L\left({p}_{1}\right)$	$L\left({p}_{2}\right)$	$n$	$c$	$L\left({p}_{1}\right)$	$L\left({p}_{2}\right)$	$n$	$c$	$L\left({p}_{1}\right)$	$L\left({p}_{2}\right)$
0.25	1.1	749	464	0.9011	0.2481	704	450	0.9001	0.2463	663	437	0.9002	0.2452	632	423	0.9	0.2494
	1.2	214	130	0.9054	0.2483	201	126	0.9044	0.2471	192	124	0.9024	0.2382	183	120	0.9008	0.2404
	1.3	106	63	0.9017	0.2388	101	62	0.9036	0.239	98	62	0.9043	0.2307	95	61	0.9032	0.2288
	1.4	67	39	0.9026	0.2334	63	38	0.908	0.2447	61	38	0.9119	0.2437	57	36	0.906	0.244
	1.5	47	27	0.9118	0.2498	46	27	0.9005	0.2177	44	27	0.9188	0.2484	44	27	0.9005	0.2042
	1.8	26	14	0.9116	0.2139	25	14	0.9184	0.2218	24	14	0.9268	0.2346	22	13	0.9202	0.2394
	2	23	12	0.9281	0.188	20	11	0.9351	0.2311	16	9	0.9174	0.2464	16	9	0.908	0.2189
0.1	1.1	1281	787	0.9013	0.099	1197	759	0.9007	0.0997	1124	735	0.9005	0.0991	1086	721	0.9004	0.0992
	1.2	363	217	0.9032	0.097	337	208	0.902	0.0989	319	203	0.9004	0.0955	317	205	0.9062	0.0956
	1.3	177	103	0.9007	0.0963	168	101	0.9024	0.0965	158	98	0.9018	0.0958	154	97	0.9022	0.0948
	1.4	111	63	0.9031	0.0957	104	61	0.9034	0.0974	101	61	0.9012	0.0884	96	59	0.9032	0.0946
	1.5	81	45	0.912	0.0955	75	43	0.9084	0.0959	71	42	0.9076	0.0941	70	42	0.9065	0.0893
	1.8	42	22	0.9236	0.0988	39	21	0.9164	0.0945	38	21	0.9118	0.0829	37	21	0.9231	0.0945
	2	34	17	0.9247	0.0795	31	16	0.9202	0.0825	28	15	0.9203	0.0924	26	14	0.9071	0.0883
0.05	1.1	–	–	–	–	1565	989	0.9011	0.0494	1473	960	0.9009	0.0487	1416	937	0.9007	0.0494
	1.2	463	275	0.9011	0.0497	437	268	0.9016	0.0495	411	260	0.9008	0.0488	397	255	0.9001	0.0486
	1.3	234	135	0.9037	0.0471	218	130	0.905	0.0495	205	126	0.9009	0.0471	197	123	0.9007	0.0481
	1.4	146	82	0.9067	0.0476	138	80	0.9048	0.0461	127	76	0.9025	0.048	125	76	0.9044	0.0468
	1.5	104	57	0.912	0.0482	101	57	0.908	0.0417	91	53	0.9026	0.0449	91	54	0.9109	0.0453
	1.8	53	27	0.913	0.0453	51	27	0.9201	0.0472	48	26	0.9081	0.0403	47	26	0.9147	0.0428
	2	39	19	0.9123	0.0454	38	19	0.9066	0.0382	37	19	0.9021	0.0324	34	18	0.9121	0.0445

(-) denotes parameters do not exist.

**Table 3:** The plan parameter when $\stackrel{\sim }{\alpha }=0.10;\beta =2$ and $a=0.50$.

$\stackrel{\sim }{\beta }$	$\mu /{\mu }_{0}$	${I}_{U}$ = 0				${I}_{U}$ = 0.02				${I}_{U}$ = 0.04				${I}_{U}$ = 0.05			
		$n$	$c$	$L\left({p}_{1}\right)$	$L\left({p}_{2}\right)$	$n$	$c$	$L\left({p}_{1}\right)$	$L\left({p}_{2}\right)$	$n$	$c$	$L\left({p}_{1}\right)$	$L\left({p}_{2}\right)$	$n$	$c$	$L\left({p}_{1}\right)$	$L\left({p}_{2}\right)$
0.25	1.1	646	108	0.9008	0.2485	617	109	0.9008	0.2443	573	107	0.901	0.2493	558	107	0.9006	0.2478
	1.2	198	31	0.9061	0.2435	181	30	0.9043	0.2496	172	30	0.901	0.2413	167	30	0.9036	0.2461
	1.3	110	16	0.9078	0.2224	103	16	0.9141	0.2354	97	16	0.9174	0.2421	94	16	0.9202	0.2482
	1.4	66	9	0.9053	0.2382	62	9	0.9085	0.2445	59	9	0.906	0.2374	58	9	0.901	0.2258
	1.5	47	6	0.9059	0.2435	45	6	0.9008	0.2305	42	6	0.9064	0.2426	41	6	0.9053	0.2392
	1.8	29	3	0.912	0.2144	27	3	0.9157	0.2236	25	3	0.921	0.2378	25	3	0.9143	0.218
	2	21	2	0.9232	0.25	20	2	0.9217	0.244	19	2	0.9209	0.2402	19	2	0.9154	0.2229
0.1	1.1	1122	183	0.9006	0.0977	1049	181	0.9008	0.0995	993	181	0.9016	0.0994	967	181	0.9009	0.0981
	1.2	327	49	0.9005	0.1	315	50	0.9044	0.0989	298	50	0.9054	0.0991	285	49	0.9003	0.0975
	1.3	174	24	0.9023	0.0954	164	24	0.9049	0.0977	155	24	0.9061	0.0983	151	24	0.9054	0.097
	1.4	117	15	0.9099	0.0942	110	15	0.9131	0.0977	105	15	0.9079	0.0901	101	15	0.9151	0.0991
	1.5	84	10	0.9094	0.0968	79	10	0.9118	0.0994	76	10	0.9034	0.0876	73	10	0.9101	0.0958
	1.8	50	5	0.9281	0.098	47	5	0.9295	0.0999	45	5	0.926	0.0929	44	5	0.9246	0.09
	2	36	3	0.908	0.0948	34	3	0.9079	0.0941	32	3	0.9091	0.0954	31	3	0.9102	0.0968
0.05	1.1	1467	237	0.9021	0.0492	1370	234	0.9004	0.0495	1297	234	0.9008	0.0492	1257	233	0.901	0.0495
	1.2	435	64	0.9016	0.0485	411	64	0.9026	0.0486	383	63	0.901	0.0494	373	63	0.9003	0.0486
	1.3	230	31	0.9042	0.0472	218	31	0.9016	0.0449	213	32	0.9044	0.0415	200	31	0.9056	0.0467
	1.4	153	19	0.908	0.0457	145	19	0.906	0.0438	142	20	0.9219	0.0473	132	19	0.9141	0.049
	1.5	112	13	0.9159	0.0498	106	13	0.915	0.0486	100	13	0.9166	0.0493	98	13	0.9127	0.0458
	1.8	64	6	0.9187	0.0468	60	6	0.9213	0.0488	57	6	0.9197	0.0468	55	6	0.9225	0.0493
	2	49	4	0.9156	0.048	46	4	0.9172	0.0493	44	4	0.9139	0.0455	44	4	0.9056	0.0381

**Table 4:** The plan parameter when $\stackrel{\sim }{\alpha }=0.10;\beta =2$ and $a=0.10$.

$\stackrel{\sim }{\beta }$	$\mu /{\mu }_{0}$	${I}_{U}$ = 0				${I}_{U}$ = 0.02				${I}_{U}$ = 0.04				${I}_{U}$ = 0.05			
		$n$	$c$	$L\left({p}_{1}\right)$	$L\left({p}_{2}\right)$	$n$	$c$	$L\left({p}_{1}\right)$	$L\left({p}_{2}\right)$	$n$	$c$	$L\left({p}_{1}\right)$	$L\left({p}_{2}\right)$	$n$	$c$	$L\left({p}_{1}\right)$	$L\left({p}_{2}\right)$
0.25	1.1	229	119	0.9104	0.2494	206	112	0.9033	0.2487	190	108	0.9003	0.2476	188	111	0.9044	0.2429
	1.2	65	32	0.9026	0.2374	60	31	0.9027	0.2425	59	32	0.9107	0.2423	56	31	0.9063	0.2399
	1.3	36	17	0.9207	0.242	35	17	0.9056	0.2027	33	17	0.9174	0.2224	32	17	0.9246	0.2366
	1.4	25	11	0.9138	0.1991	23	11	0.9348	0.2492	22	11	0.9346	0.2423	18	9	0.908	0.2397
	1.5	17	7	0.9043	0.197	16	7	0.911	0.2072	15	7	0.9202	0.2244	15	7	0.9089	0.194
	1.8	11	4	0.9334	0.1844	11	4	0.9184	0.1417	10	4	0.9332	0.1743	9	4	0.9539	0.2495
	2	10	3	0.9142	0.109	10	4	0.9742	0.2217	9	3	0.9157	0.1047	9	4	0.9786	0.2495
0.1	1.1	371	189	0.9004	0.0992	352	188	0.9035	0.0994	324	181	0.9003	0.0995	317	181	0.9002	0.0966
	1.2	109	52	0.902	0.0956	104	52	0.9022	0.0919	99	52	0.9087	0.0946	97	52	0.9059	0.0888
	1.3	56	25	0.9013	0.0915	55	26	0.9151	0.0951	51	25	0.9014	0.0838	51	26	0.9224	0.0982
	1.4	38	16	0.9117	0.0872	36	16	0.9163	0.0896	35	16	0.9014	0.0689	35	17	0.935	0.0958
	1.5	30	0	0.9254	0.0808	28	12	0.9365	0.0949	27	12	0.9311	0.0818	24	11	0.9294	0.0987
	1.8	15	5	0.9172	0.0839	14	5	0.924	0.0916	14	5	0.9053	0.0628	13	5	0.9247	0.0872
	2	13	4	0.9343	0.0759	13	4	0.9196	0.0525	12	4	0.9288	0.0614	9	3	0.9085	0.0904
0.05	1.1	494	250	0.9061	0.0496	462	245	0.9056	0.0495	431	239	0.9019	0.0476	416	236	0.9038	0.0488
	1.2	146	69	0.9129	0.0497	138	68	0.9018	0.042	129	67	0.9132	0.0486	119	63	0.9016	0.0485
	1.3	73	32	0.9021	0.0452	69	32	0.9123	0.0499	66	32	0.9102	0.0454	65	32	0.901	0.038
	1.4	49	20	0.9037	0.0389	44	19	0.9066	0.046	42	19	0.9061	0.043	41	19	0.9071	0.0423
	1.5	38	15	0.934	0.0462	37	15	0.9201	0.0328	35	15	0.9255	0.0342	30	13	0.9061	0.0393
	1.8	22	7	0.9189	0.0277	21	7	0.917	0.0251	20	7	0.9168	0.0234	18	7	0.9474	0.0496
	2	20	6	0.9487	0.0243	18	6	0.9616	0.0376	17	6	0.9633	0.0381	16	5	0.9109	0.0161

**Table 5:** The plan parameter when $\stackrel{\sim }{\alpha }=0.10;\beta =3$ and $a=0.50$.

$\stackrel{\sim }{\beta }$	$\mu /{\mu }_{0}$	${I}_{U}$ = 0				${I}_{U}$ = 0.02				${I}_{U}$ = 0.04				${I}_{U}$ = 0.05			
		$n$	$c$	$L\left({p}_{1}\right)$	$L\left({p}_{2}\right)$	$n$	$c$	$L\left({p}_{1}\right)$	$L\left({p}_{2}\right)$	$n$	$c$	$L\left({p}_{1}\right)$	$L\left({p}_{2}\right)$	$n$	$c$	$L\left({p}_{1}\right)$	$L\left({p}_{2}\right)$
0.25	1.1	639	49	0.903	0.246	580	48	0.9005	0.2473	538	48	0.9007	0.2471	518	48	0.9019	0.2491
	1.2	190	13	0.9013	0.2489	188	14	0.9116	0.2466	174	14	0.9129	0.249	157	13	0.9023	0.2497
	1.3	113	7	0.9187	0.2444	100	7	0.9208	0.2497	97	7	0.9188	0.2436	82	6	0.9002	0.2494
	1.4	73	4	0.916	0.2449	68	4	0.9142	0.2395	63	4	0.9144	0.2396	60	4	0.9176	0.2483
	1.5	59	3	0.9323	0.2491	55	3	0.9309	0.2436	51	3	0.9308	0.243	49	3	0.9314	0.2446
	1.8	32	1	0.9156	0.2306	29	1	0.9185	0.2401	27	1	0.9178	0.2372	26	1	0.9178	0.2371
	2	31	1	0.9539	0.2461	29	1	0.953	0.2401	27	1	0.9526	0.2372	26	1	0.9526	0.2371
0.1	1.1	1097	81	0.9002	0.0967	1014	81	0.9032	0.0995	930	80	0.9002	0.0987	896	80	0.9007	0.099
	1.2	341	22	0.9051	0.0988	316	22	0.9044	0.0976	293	22	0.9045	0.0973	282	22	0.9054	0.0982
	1.3	193	11	0.9151	0.0966	165	10	0.9002	0.0993	153	10	0.9002	0.0988	159	11	0.9169	0.0982
	1.4	122	6	0.9035	0.0972	126	7	0.9289	0.0982	104	6	0.9059	0.0999	101	6	0.9028	0.0953
	1.5	92	4	0.9075	0.0991	85	4	0.9079	0.0995	79	4	0.9071	0.0979	76	4	0.9074	0.0983
	1.8	61	2	0.9345	0.0988	57	2	0.9329	0.0948	53	2	0.9323	0.0932	51	2	0.9325	0.0933
	2	45	1	0.9113	0.0945	41	1	0.9135	0.0988	38	1	0.9134	0.0982	37	1	0.9117	0.0947
0.05	1.1	1428	104	0.9023	0.0499	1323	104	0.9015	0.0491	1227	104	0.9017	0.049	1171	103	0.9008	0.0498
	1.2	446	28	0.9039	0.0493	414	28	0.9016	0.0474	384	28	0.9014	0.047	369	28	0.9038	0.0485
	1.3	239	13	0.9032	0.0496	221	13	0.9037	0.0498	205	13	0.9034	0.0493	198	13	0.9019	0.048
	1.4	167	8	0.9116	0.0483	154	8	0.913	0.0493	143	8	0.9123	0.0484	138	8	0.9116	0.0477
	1.5	121	5	0.903	0.0491	112	5	0.9028	0.0487	104	5	0.9021	0.0479	100	5	0.9027	0.0483
	1.8	72	2	0.9037	0.0492	67	2	0.9023	0.0476	62	2	0.9025	0.0476	60	2	0.9014	0.0464
	2	72	2	0.954	0.0492	67	2	0.9533	0.0476	64	2	0.9496	0.0408	61	2	0.9509	0.0428

**Table 6:** The plan parameter when $\stackrel{\sim }{\alpha }=0.10;\beta =3$ and $a=0.10$.

$\stackrel{\sim }{\beta }$	$\mu /{\mu }_{0}$	${I}_{U}$ = 0				${I}_{U}$ = 0.02				${I}_{U}$ = 0.04				${I}_{U}$ = 0.05			
		$n$	$c$	$L\left({p}_{1}\right)$	$L\left({p}_{2}\right)$	$n$	$c$	$L\left({p}_{1}\right)$	$L\left({p}_{2}\right)$	$n$	$c$	$L\left({p}_{1}\right)$	$L\left({p}_{2}\right)$	$n$	$c$	$L\left({p}_{1}\right)$	$L\left({p}_{2}\right)$
0.25	1.1	106	50	0.9023	0.2486	104	52	0.9016	0.2298	92	49	0.9035	0.2472	90	51	0.9085	0.2406
	1.2	35	15	0.904	0.2157	35	16	0.9109	0.2045	33	16	0.9099	0.1972	28	14	0.9008	0.2192
	1.3	18	7	0.9041	0.216	17	7	0.9007	0.2045	16	7	0.9001	0.1986	15	7	0.9183	0.2385
	1.4	14	5	0.9223	0.1919	13	5	0.9256	0.1969	10	4	0.9054	0.2146	12	5	0.921	0.1773
	1.5	9	3	0.9267	0.2358	9	3	0.9081	0.1803	8	3	0.9238	0.218	8	3	0.9149	0.1903
	1.8	7	2	0.9628	0.2115	7	2	0.9547	0.1651	4	1	0.9112	0.2115	4	1	0.9052	0.1915
	2	5	1	0.9391	0.176	5	1	0.93	0.1405	4	1	0.9486	0.2115	4	1	0.9449	0.1915
0.1	1.1	183	84	0.9032	0.0987	168	82	0.9003	0.0976	158	82	0.9043	0.0978	159	85	0.9093	0.0926
	1.2	56	23	0.9011	0.0895	52	23	0.9117	0.0991	47	22	0.9037	0.0959	48	23	0.9008	0.0805
	1.3	32	12	0.9215	0.0892	28	11	0.9038	0.0836	29	12	0.9028	0.0617	23	10	0.9023	0.0983
	1.4	21	7	0.9149	0.0809	17	6	0.9011	0.0949	18	7	0.9255	0.0905	18	7	0.9114	0.0692
	1.5	17	5	0.9132	0.0617	16	5	0.9105	0.0566	14	5	0.9342	0.087	14	5	0.9238	0.0685
	1.8	12	3	0.9596	0.0644	11	3	0.9621	0.0688	10	3	0.9658	0.0775	8	2	0.9134	0.0566
	2	11	2	0.9394	0.0284	10	2	0.943	0.0313	8	2	0.9628	0.0686	5	1	0.9144	0.0953
0.05	1.1	236	107	0.9001	0.0489	226	109	0.9015	0.0447	212	109	0.9113	0.0482	195	103	0.9006	0.0483
	1.2	72	29	0.9005	0.0452	65	28	0.9012	0.0489	61	28	0.9034	0.0479	59	28	0.9072	0.0491
	1.3	39	14	0.9049	0.0424	36	14	0.9152	0.0483	34	14	0.9111	0.0426	33	14	0.9104	0.0408
	1.4	28	9	0.9142	0.0352	26	9	0.9179	0.0362	24	9	0.9255	0.0402	21	8	0.9098	0.0437
	1.5	20	6	0.9307	0.0486	20	6	0.9042	0.026	18	6	0.9206	0.0351	17	6	0.93	0.0423
	1.8	14	3	0.9321	0.0244	12	3	0.9486	0.0409	11	3	0.9519	0.0441	11	3	0.946	0.0345
	2	11	2	0.9394	0.0284	10	2	0.943	0.0313	9	2	0.9482	0.0367	9	2	0.9432	0.0292

## Comparative study

In this section, the efficiency of the proposed plan is discussed in terms of sample size. The smaller the sample size means that less cost is needed for testing the hypothesis about the daily average wind speed. Note here that the proposed sampling plan is the generalization of the plan under classical statistics when no uncertainty/indeterminacy is found in recording the daily average wind speed. The proposed sampling plan reduces to the existing sampling plan when ${I}_{N}$=0. The first column in Tables 1, 2, 3, 4, 5 and 6 presents the plan parameters under the classical statistics. From Tables 1, 2, 3, 4, 5 and 6, it can be noted that the values of the sample size required for testing ${H}_{0}:\mu ={\mu }_{0}$ decreasing as the indeterminacy parameter ${I}_{N}$ increases. For example, when $\mu /\mu_{0} = 1.1$ and $a = 0.5$ from Table 1, it can be seen that $n = 1143$ from the plan under classical statistics and $n = 1010$ for the proposed sampling plan when $I_{N} = 0.05$. From the study, it is concluded that the proposed plan under indeterminacy is efficient in sample size as compared to the existing sampling plan under classical statistics. Therefore, the application of the proposed plan for testing the null hypothesis ${H}_{0}:\mu ={\mu }_{0}$ requires a smaller sample as compared to the existing plan. The meteorologist can apply the proposed plan under uncertainty with fewer effort and time.

## Application for wind speed data

The application of the proposed sampling plan will be discussed using wind speed data. The wind speed is a big and important source of energy. Due to the randomness and uncertainty, the wind speed data follows the statistical distribution under neutrosophic statistics. The meteorologists are interested to see the daily average wind speed under indeterminacy. The average wind speed data of Cairo city is taken from^40^ and shown in Table 7. It is found that the wind speed data follows the Weibull distribution with shape parameter $\widehat{\beta }=$ 2.7857 and scale parameter $\alpha =8.05$. The plan parameters for this shape parameter are shown in Tables 8 and 9. For the proposed plan, the shape parameter is ${\widehat{\beta }}_{N}=(1+0.02)\times 2.7857 \approx 3$ when $I_{U} = 0.02$. Suppose that meteorologists are interested to test $H_{0} :\mu = 7.15$ with the aid of the proposed sampling plan when $I_{U} = 0.02$, $\tilde{\alpha } = 0.10$, $\mu /\mu_{0} = 1.3$, $a = 0.5$ and $\tilde{\beta } = 0.25$. From Table 5, it can be noted that $n = 100$ and $c = 7$. The proposed sampling plan will be implemented as: accept the null hypothesis ${H}_{0}:\mu =7.15$ if average daily speed in 7 days is more than equal to 7.15mph. From the data, it can be noted average daily wind speed is more than equal to 7.15mph in more than 7 days, therefore, the claim about the average daily wind speed ${H}_{0}:\mu =7.15$ will be accepted. From the real example, it is concluded that the proposed sampling will be helpful to check the daily average wind speed.

**Table 7:** The daily average wind speed data.

2.7	4.2	4.9	5.4	5.7	6.8	7.5	8.6	9.5	11.1
3.1	4.2	4.9	5.4	5.8	6.8	7.6	8.7	9.6	11.3
3.2	4.3	4.9	5.4	5.8	6.8	7.6	8.8	9.8	12
3.2	4.3	5	5.5	6	6.8	7.7	8.9	9.8	12.2
3.3	4.3	5	5.5	6.1	6.9	7.8	9.3	9.9	12.4
3.5	4.5	5.1	5.6	6.3	7.1	7.9	9.3	10	12.5
3.5	4.7	5.2	5.6	6.4	7.3	8	9.3	10.1	13.3
3.8	4.7	5.2	5.6	6.6	7.3	8	9.4	10.3	13.8
3.8	4.8	5.3	5.7	6.7	7.3	8.2	9.4	10.6	14.4
3.8	4.9	5.4	5.7	6.7	7.4	8.2	9.4	10.7	14.7

**Table 8:** The plan parameter when $\stackrel{\sim }{\alpha }=0.10;\beta =2.7857$ and $a=0.50$.

$\stackrel{\sim }{\beta }$	$\mu /{\mu }_{0}$	${I}_{U}$ = 0				${I}_{U}$ = 0.02				${I}_{U}$ = 0.04				${I}_{U}$ = 0.05			
		$n$	$c$	$L\left({p}_{1}\right)$	$L\left({p}_{2}\right)$	$n$	$c$	$L\left({p}_{1}\right)$	$L\left({p}_{2}\right)$	$n$	$c$	$L\left({p}_{1}\right)$	$L\left({p}_{2}\right)$	$n$	$c$	$L\left({p}_{1}\right)$	$L\left({p}_{2}\right)$
0.25	1.1	630	57	0.9032	0.2455	585	57	0.9057	0.2499	536	56	0.9029	0.2494	518	56	0.9022	0.2476
	1.2	195	16	0.9093	0.2476	171	15	0.9005	0.2495	169	16	0.9093	0.2463	163	16	0.9101	0.2479
	1.3	108	8	0.9143	0.2407	100	8	0.912	0.2344	93	8	0.9167	0.2457	90	8	0.9159	0.2431
	1.4	74	5	0.9223	0.2418	69	5	0.9215	0.2389	64	5	0.9227	0.2418	62	5	0.9217	0.2387
	1.5	51	3	0.9101	0.2393	47	3	0.9123	0.2449	45	3	0.9051	0.225	42	3	0.9138	0.2482
	1.8	40	2	0.9532	0.2253	36	2	0.9568	0.244	34	2	0.9552	0.2347	22	1	0.9012	0.245
	2	27	1	0.9376	0.2347	25	1	0.938	0.2358	23	1	0.9392	0.2408	22	1	0.9402	0.245
0.1	1.1	1064	93	0.9002	0.0992	1000	94	0.902	0.0983	922	93	0.9005	0.0986	890	93	0.9014	0.0993
	1.2	336	26	0.9083	0.0992	302	25	0.9006	0.0989	281	25	0.9016	0.0995	281	26	0.9086	0.0983
	1.3	176	12	0.9039	0.0983	164	12	0.9027	0.0964	152	12	0.9054	0.0993	147	12	0.9045	0.0979
	1.4	116	7	0.9031	0.0988	108	7	0.9024	0.0975	101	7	0.9005	0.0945	97	7	0.9028	0.0973
	1.5	91	5	0.9161	0.0997	85	5	0.9145	0.0968	79	5	0.915	0.0972	76	5	0.9161	0.0987
	1.8	52	2	0.9121	0.0982	49	2	0.9092	0.0927	45	2	0.9119	0.097	44	2	0.9092	0.0921
	2	52	2	0.956	0.0982	49	2	0.9544	0.0927	45	2	0.9559	0.097	44	2	0.9544	0.0921
0.05	1.1	1404	121	0.9011	0.049	1295	120	0.9006	0.0496	1216	121	0.9023	0.0491	1165	120	0.9009	0.0493
	1.2	438	33	0.9061	0.049	396	32	0.9009	0.0499	369	32	0.9004	0.0493	356	32	0.9013	0.0497
	1.3	228	15	0.9001	0.0495	223	16	0.9138	0.0495	209	16	0.9099	0.0461	201	16	0.9123	0.0478
	1.4	155	9	0.9029	0.0482	144	9	0.9032	0.0481	134	9	0.9034	0.048	129	9	0.9047	0.0489
	1.5	116	6	0.9062	0.0498	108	6	0.9055	0.0489	101	6	0.9037	0.0471	97	6	0.9057	0.0486
	1.8	76	3	0.9318	0.048	71	3	0.9307	0.0464	66	3	0.9309	0.0464	63	3	0.9331	0.0491
	2	62	2	0.9325	0.0464	57	2	0.9341	0.0485	54	2	0.9312	0.0442	52	2	0.9315	0.0445

**Table 9:** The plan parameter when $\stackrel{\sim }{\alpha }=0.10;\beta =2.7857$ and $a=0.10$.

$\stackrel{\sim }{\beta }$	$\mu /{\mu }_{0}$	Alpha = 0.10; b = 2.7857; a = 1.0															
		${I}_{U}$ = 0				${I}_{U}$ = 0.02				${I}_{U}$ = 0.04				${I}_{U}$ = 0.05			
		$n$	$c$	$L\left({p}_{1}\right)$	$L\left({p}_{2}\right)$	$n$	$c$	$L\left({p}_{1}\right)$	$L\left({p}_{2}\right)$	$n$	$c$	$L\left({p}_{1}\right)$	$L\left({p}_{2}\right)$	$n$	$c$	$L\left({p}_{1}\right)$	$L\left({p}_{2}\right)$
0.25	1.1	123	59	0.9028	0.2445	112	57	0.9023	0.2498	104	56	0.9022	0.2494	103	57	0.9045	0.2448
	1.2	36	16	0.9055	0.2485	34	16	0.9043	0.2413	34	17	0.9127	0.234	31	16	0.9116	0.2492
	1.3	26	11	0.9475	0.2295	23	10	0.9262	0.1966	19	9	0.9334	0.2499	17	8	0.9075	0.2198
	1.4	16	6	0.9251	0.1925	15	6	0.9266	0.1922	14	6	0.9306	0.1983	13	6	0.9464	0.2486
	1.5	12	4	0.9156	0.1663	11	4	0.9237	0.1825	10	4	0.9343	0.2093	10	4	0.9256	0.1807
	1.8	7	2	0.9475	0.2028	7	2	0.937	0.1594	6	2	0.9518	0.2141	6	2	0.9473	0.1911
	2	5	1	0.9191	0.1694	4	1	0.9407	0.2481	4	1	0.9325	0.208	4	1	0.928	0.189
0.1	1.1	214	100	0.9024	0.0925	201	100	0.9121	0.0997	179	94	0.901	0.0973	174	94	0.9032	0.0971
	1.2	66	28	0.9087	0.0883	62	28	0.9117	0.0887	56	27	0.9168	0.0999	53	26	0.9019	0.0883
	1.3	34	13	0.9037	0.0843	32	13	0.9033	0.081	30	13	0.9073	0.0822	29	13	0.911	0.0847
	1.4	23	8	0.91	0.0812	21	8	0.924	0.0985	20	8	0.9188	0.0868	20	8	0.9034	0.066
	1.5	19	6	0.9184	0.0652	17	6	0.9359	0.0891	17	6	0.9127	0.054	16	6	0.9252	0.067
	1.8	11	3	0.9544	0.0952	11	3	0.9428	0.0651	10	3	0.9489	0.0749	10	3	0.943	0.0613
	2	9	2	0.9476	0.076	9	2	0.9372	0.0529	8	2	0.9456	0.0664	8	2	0.9406	0.0552
0.05	1.1	268	124	0.9001	0.0495	259	127	0.901	0.0441	240	125	0.9091	0.0487	228	122	0.9052	0.0483
	1.2	84	35	0.9081	0.0452	77	34	0.9028	0.0439	72	34	0.9138	0.049	68	33	0.9101	0.0494
	1.3	45	17	0.9159	0.0451	42	17	0.9215	0.0476	40	17	0.9136	0.039	39	17	0.9106	0.0358
	1.4	29	10	0.921	0.0493	28	10	0.9056	0.0354	26	10	0.9134	0.0387	25	10	0.9187	0.0416
	1.5	23	7	0.9125	0.0342	21	7	0.9241	0.0418	20	7	0.9186	0.0352	19	7	0.9275	0.0416
	1.8	13	3	0.9201	0.0366	12	3	0.9235	0.0384	11	3	0.929	0.0423	11	3	0.9212	0.0334
	2	10	2	0.9305	0.045	13	3	0.9575	0.0222	9	2	0.9252	0.0353	9	2	0.9186	0.0283

## Concluding remarks

The time truncated plan for the Weibull distribution under the indeterminacy was presented. The plan parameters of the proposed plan were determined by fixing the indeterminacy parameter. The plan parameters were given for various values of indeterminacy parameters, shape parameter, and scale parameter. Several tables for the application of the proposed plan are given. The application of the proposed plan was given with the help of daily average wind speed. The testing of the hypothesis was done to test the average daily wind speed. From the study, it is concluded that the indeterminacy parameter plays a significant role in fixing the plan parameters. The less sample size is needed as the indeterminacy parameter increased. In addition, it is found that the proposed plan is efficient than the existing sampling plan in terms of sample size. To save time, efforts, and energy, it is recommended to apply the proposed plan for testing the average wind speed. The proposed plan can be applied in metrology, oceanography, and thermodynamics. The proposed plan can be applied for testing big data from oceanography as future research. By following^41,42^, the software for goodness of fit tests using the npdf in Eq. (1) can be developed as future research.

## Data Availability

The data is given in the paper.
